# Association between neighborhood disadvantage and chronic hepatitis B in the central Puget Sound region of Washington, 2018 to 2023

**DOI:** 10.1371/journal.pone.0349563

**Published:** 2026-06-15

**Authors:** Grace Dickel, Kristine Lan, Christine Khosropour, H. Nina Kim

**Affiliations:** 1 Department of Epidemiology, University of Washington School of Public Health, Seattle, Washington, United States of America; 2 Division of Allergy and Infectious Disease, Department of Medicine, University of Washington School of Medicine, Seattle, Washington, United States of America; Emory University, UNITED STATES OF AMERICA

## Abstract

**Background:**

Chronic Hepatitis B (CHB) disproportionately affects immigrant and socioeconomically marginalized populations in the United States. Neighborhood-level metrics such as the Area Deprivation Index (ADI) may provide insight into structural and geographic indicators of CHB risk.

**Methods and findings:**

We used electronic health record data from the University of Washington Medicine system to assess the association between neighborhood-level social deprivation and a positive CHB status among adults aged ≥18 with at least one clinical encounter between 2018 and 2023. We conducted a case-control study where CHB cases were identified via positive hepatitis B surface antigen or detectable HBV DNA. Comparators were selected from patients who had a complete blood count (CBC) or chemistry panel testing as a general marker of healthcare engagement to ensure comparable opportunity for diagnosis. The comparators were then frequency matched to cases by care setting and calendar year quarter of medical encounter. Geocoded patient billing addresses were linked to census block groups to confer ADI scores, which were analyzed in 2-, 5-, and 10-level categories. Finer ADI stratification better captured gradients in neighborhood disadvantage and revealed the clearest trends; results from the 10-level ADI categories are noted here. Logistic regression models adjusted for age, sex, race, ethnicity, and insurance status were used to estimate odds ratios (OR) for CHB cases across ADI categories. The final study cohort included 5,729 CHB cases and 6,143 comparators. CHB was more common among individuals identifying as Asian, Black, or Native Hawaiian/Pacific Islander, and those with public or no insurance. Adjusted models showed higher odds of a CHB positive test among residents of more disadvantaged neighborhoods, with the highest ADI category associated with twice the odds of CHB compared to the least disadvantaged category (adjusted OR = 2.07, 95% CI: 1.51–2.82), with an overall upward trend across ADI levels.

**Conclusions:**

Neighborhood-level disadvantage was associated with higher odds of a CHB positive test when using granular stratification of ADI and after adjusting for individual-level factors. These findings support the integration of place-based metrics to inform targeted public health initiatives, including community-responsive education, and geographically focused hepatitis B screening and linkage-to-care efforts in socially disadvantaged neighborhoods.

## Introduction

Chronic hepatitis B (CHB) is a leading cause of cirrhosis, liver failure, and hepatocellular carcinoma (HCC), accounting for more than half of all HCC cases worldwide [1–3]. An estimated 254 million individuals live with CHB globally, many of whom remain undiagnosed due to the asymptomatic nature of the infection, and approximately one in five will develop severe liver-related complications over their lifetime. [1]. In the United States (U.S.), about 640,000 individuals are living with CHB, the highest burden from people born in hepatitis B-endemic regions, including parts of Southeast Asia, Africa, and the Pacific Islands [1,4–8]. Among non-US-born populations, infection is most commonly acquired through perinatal transmission [4,8]. Foreign-born persons also experience disproportionate barriers to hepatitis B screening, vaccination, and treatment due to structural inequities, limited access to care, and language and cultural barriers [9–12].

The burden of CHB and its complications is particularly pronounced in immigrant and low-income communities. The Puget Sound region of Washington State is home to a large and diverse immigrant population, many of whom originate from hepatitis B-endemic regions, making it a critical setting in which to examine disparities in CHB burden [1,4,13].

While individual–level factors such as race, ethnicity, sex, and country of origin are known to be associated with CHB prevalence [2,4,5], area-level socioeconomic measures may provide additional insight into broader structural determinants of disease distribution. The Area Deprivation Index (ADI) is a validated, composite measure of neighborhood-level socioeconomic disadvantage based on income, employment, education, and housing characteristics derived from the U.S. Census data [6]. Indices such as the ADI may be preferable to individual metrics alone because they reflect broader neighborhood conditions that shape access to health-promoting resources and structural factors, such as differences in access to primary care services, availability of culturally and linguistically appropriate healthcare, and neighborhood-level barriers to preventative screening. ADI has been associated with adverse outcomes across multiple health conditions, including cancer care, diabetes, COVID-19, and autoimmune diseases such as diabetes and discoid lupus [7–10]. Neighborhood income has been shown to be inversely associated with hepatitis B and C viral load [11], but the relationship between neighborhood-level deprivation and the distribution of CHB in the United States has not been directly examined. In addition to capturing structural disadvantage, area-level indices such as ADI may also approximate unmeasured social and cultural factors that are often unavailable in clinical or administrative datasets. However, immigrant and ethnically minoritized populations frequently reside in geographically clustered communities shaped by socioeconomic and cultural characteristics. Thus, neighborhood-level deprivation may indirectly capture contextual risk factors, including country of origin, that are frequently missing from clinical data [12–14].

Understanding how area-level disadvantage shapes the burden of CHB is particularly important given the lifelong nature of the infection, its concentration in marginalized populations, and its risk of severe complications such as cirrhosis and HCC. The primary aim of this study was to evaluate the association between neighborhood-level social deprivation, as measured by the ADI, and chronic hepatitis B among adults receiving care in the Puget Sound region. We hypothesized that individuals residing in areas of higher social deprivation would have higher odds of CHB compared to individuals residing in areas of lower social deprivation, independent of individual-level demographic factors. Findings from this work are intended to inform targeted public health interventions and to advance health equity in liver health.

## Methods

### Study design and population

We queried the electronic health record (EHR) data of all individuals aged 18 years or older who had at least one encounter with the University of Washington Medicine healthcare system, one of the largest medical networks in the Puget Sound region, serving over 1.6 million patients annually,‌‌ between January 1, 2018, and December 31, 2023 [15]. We identified patients as having CHB if they had a positive hepatitis B surface-antigen test or a detectable level of hepatitis B virus (HBV) recorded in their EHR. We generated a comparator group by selecting patients who had a complete blood count (CBC) or a chemistry panel done during the study period but who had no positive hepatitis B surface antigen test or detectable level of HBV during that time. The comparator group was not selected based on hepatitis B screening history (i.e., a negative HBV test) to ensure it reflected a general care-seeking population, not specifically enriched or excluded based on hepatitis B screening history. CBCs and chemistry panels were used on the basis for comparator selection because they are among the most commonly ordered laboratory studies across a wide range of clinical settings and indications. These CBCs and chemistry panels serve as a relatively neutral marker of healthcare engagement and access to laboratory services rather than being linked to liver disease, infectious disease risk, or hepatitis B-specific evaluation. The comparator selection approach was intended to minimize selection bias that could arise from conditions on hepatitis B- or liver-related testing, while ensuring that both CHB cases and comparators had comparable opportunities for laboratory-based diagnosis within the health system. We frequency-matched comparator patients to those with CHB by the department where they received care and by calendar year quarter (e.g., January-March 2022). The comparator group was intentionally oversampled by 10% for each quarter to ensure statistical power, yielding a final sample size of 6,955 individuals in the comparator group and 6,500 CHB cases. Clinical data were extracted from the University of Washington data warehouse on February 3, 2025, by a designated data analyst who used structured query language (SQL) programming to identify and retrieve the required variables. The analyst removed all direct identifiers and applied standardized deidentification procedures before preparing the finalized analytic dataset for researcher access.

### Data measures

The primary exposure of interest was neighborhood-level social deprivation as defined by the Neighborhood Atlas [6]. For this study, “neighborhood” was defined as census block groups, which are statistical divisions of census tracts that contain between 600 and 3,000 people [16]. We determined the census block group for each patient in the study population by joining the geocoded patient billing address available in the EHR (assumed to represent residential address) with the 2020 census block group shapefiles from the Washington State Office of Financial Management [16]. Billing addresses were used because they were the most consistently available and systematically geocoded address field in the EHR. The neighborhood level of social deprivation was approximated using the 2022 Area Deprivation Index (ADI) created by the Neighborhood Atlas [6]. The ADI is a validated, composite measure of neighborhood-level socioeconomic disadvantage derived from 17 indicators from the American Community Survey. These indicators span multiple domains, including income, educational attainment, employment, and housing quality, which are aggregated at the U.S. census block group level. A complete list of ADI indicators is provided in S1 Table [17] between 1 and 10, with higher scores indicating more social disadvantage. We categorized the ADI WA state decile rank split into 5 groups by collapsing decile groups into five categories as follows: 1–2, 3–4, 5–6, 7–8, and 9–10. The 5-level categorization was included as an intermediate sensitivity analysis between binary and decile-based categorizations, with a priori consideration of potential small cell counts in the 10-level analysis.

Our outcome of interest was diagnosed CHB defined as a positive hepatitis B surface-antigen test or a detectable level of hepatitis B virus (HBV).

We also abstracted data from EHR on characteristics that we identified *a priori* as being potential confounders of the relationship between ADI and CHB. These measures included: age (years), sex assigned at birth (male, female), race (American Indian Alaska Native (AIAN), Asian, Black or African American, Multi-Racial, Native Hawaiian or Pacific Islanders (NHPI), and White), ethnicity (Hispanic, or non-Hispanic), and insurance status. For descriptive analysis, the distribution of the original uncollapsed insurance categories was used to provide a full view of the study population. For all inferential analyses, the insurance variable was collapsed into: Commercial, Commercial and Public, Medicaid, Medicare, Medicaid/ Medicare, Self-Pay, and Other. Due to the small proportion of individuals in the Commercial and Public, Medicare and Medicaid, Self-Pay, and Other, we reassigned groups (individuals on Medicare, Medicaid, or both Medicare and Medicaid were grouped into “Public” insurance, and individuals with Self-Pay were collapsed into “Other” insurance).

This study was approved by the University of Washington Human Subjects Division, STUDY00005397.

### Statistical analysis

We assessed the distribution of the demographic and clinical characteristics of individuals with CHB compared to individuals in the comparator group. Mean and standard deviation were reported for continuous variables and counts with percentages were reported for categorical variables.

We also assessed the distribution of CHB geographically using QGIS 3.40.5-Bratislava (https://qgis.org). Although the analyzed study population includes individuals from across Washington state, only census block groups within the Puget Sound region were included in the distribution maps to protect patient confidentiality, given the sparseness of residence outside the Puget Sound region.

For our primary analysis, we reported the odds ratio of CHB cases across the 5 ADI levels and visually displayed the distribution. We assessed the association between ADI and the odds of CHB using logistic regression to estimate odds ratios (OR) and 95% confidence intervals (CI), adjusted for age, sex, race, ethnicity, and insurance status. We used participants from the ADI category 1 (lowest level of neighborhood-level social deprivation) as the reference group. Missing data for covariates, including race and ethnicity, were limited (5.6% and 5.8%, respectively), Analyses were conducted using a complete case approach.

We conducted a sensitivity analysis to assess the association between ADI and CHB using different groupings of ADI scores by categorizations of ten (keeping the original 1–10 categorization) and two ADI levels (grouping ADI categories 1–5 vs. 6–10). We examined the association between ADI and the odds of CHB using logistic regression, adjusted for age, race, sex, ethnicity, and insurance status. All data analyses were performed using R version 2024.12.0 + 467 (R Foundation for Statistical Computing, Vienna, Austria).

## Results

**Fig 1:**
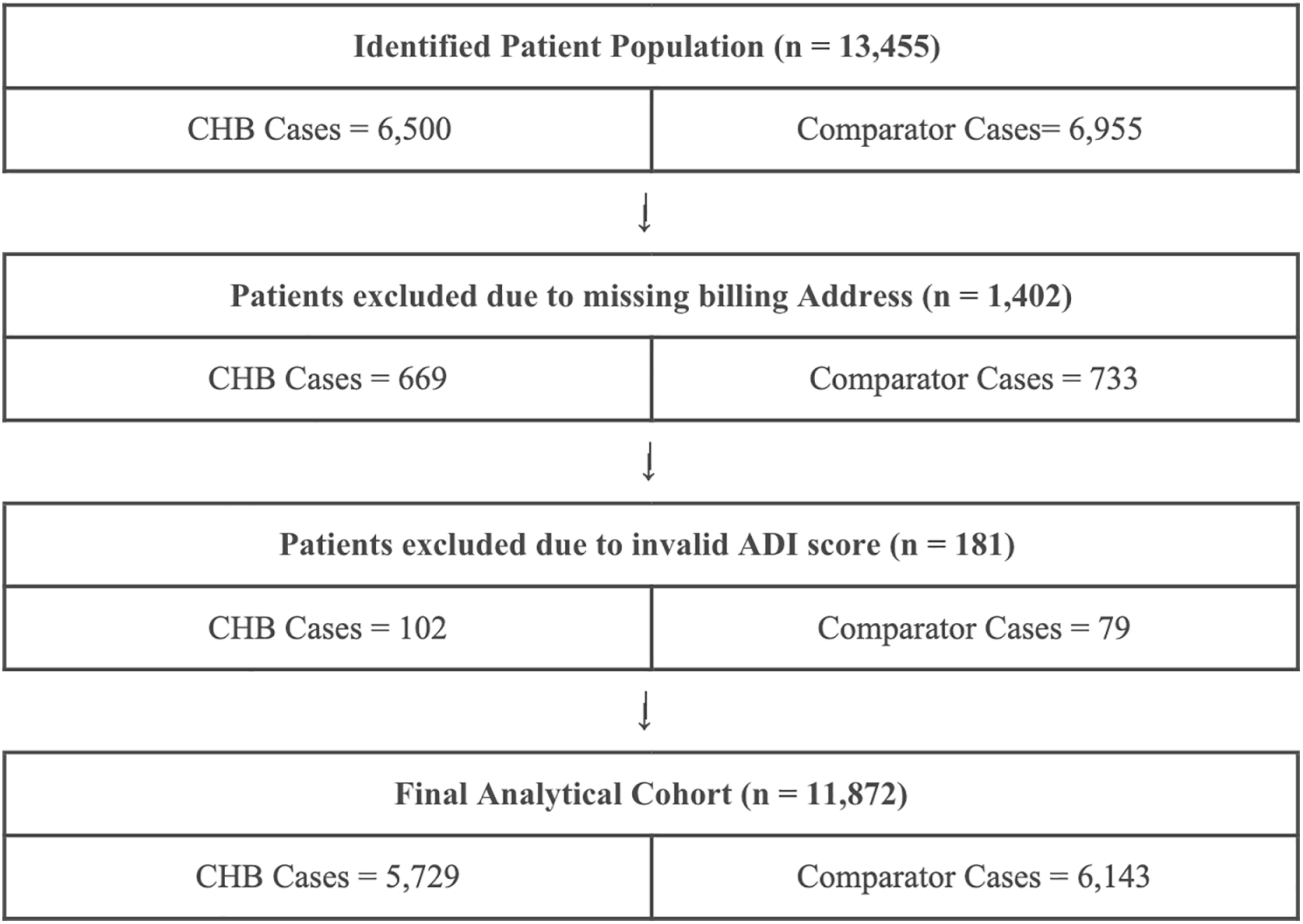
Flow diagram of cohort identification, exclusions, and final analytic group. Of 13.455 individuals initially identified, exclusions were applied for missing billing address and inability to calculate ADI score, resulting in a final analytic cohort of 11,872 individuals.

We initially identified a total of 13,455 individuals, comprising 6,500 individuals with CHB and 6,955 individuals in the comparator group. Of these, 1,402 individuals (669 with CHB and 733 comparators) were excluded due to missing geographic information required for census block group determination. An additional 181 participants (102 with CHB and 79 comparators) were excluded due to the inability to calculate the ADI score as a result of missing index components. Thus, the final analytical cohort comprised 11,872 individuals, including 5,729 individuals with CHB and 6,143 individuals in the comparator group, as seen in Fig 1.

The distribution of demographic, socioeconomic, and health characteristics of the CHB and comparator groups is displayed in Table 1. The two groups had a similar mean age (56 vs. 55 years). About 60% of individuals with CHB were male, compared to 53% of the comparator group. A higher percentage of individuals with CHB were identified as Asian (34.4% vs. 11.7%), Black or African American (22.9% vs. 12.1%), or Native Hawaiian/Pacific Islander (2.4% vs. 0.9%). Additionally, a lower proportion of individuals with CHB were Hispanic compared to the comparator group (5.1% vs. 10.5%). The distribution of insurance coverage also differed between the two groups. A higher proportion of individuals with CHB were insured by Medicaid or self-pay, and a lower proportion were insured by commercial insurance or Medicare.

**Table 1:** Demographic, socioeconomic, and health characteristics among study participants from the University of Washington Medicine, 2018-2023, segregated by history of chronic Hepatitis B.

Characteristic	History of Chronic Hepatitis B N = 5,729	No History of Chronic Hepatitis B Comparator N = 6,143
**Age** (Mean (SD))	56 (14.5)	55 (17.6)
**Sex Assigned at Birth** (N(%))		
Female^+^	2,274 (39.7%)	2,909 (47.4%)
Male^+^	3,454 (60.4%)	3,234 (52.6%)
**Race** (N(%))		
AI/AN*^+^	59 (1.0%)	107 (1.7%)
Asian^+^	1,973 (34.4%)	721 (11.7%)
Black or African American^+^	1,313 (22.9%)	743 (12.1%)
Multi-Racial	93 (1.6%)	117 (1.9%)
NHPI**^+^	136 (2.4%)	55 (0.9%)
White^+^	1,810 (31.6%)	4,042 (65.8%)
Other^+^	25 (0.4%)	42 (0.7%)
Missing	320 (5.6%)	316 (5.1%)
**Ethnicity (Hispanic)** (N(%))		
Hispanic^+^	300 (5.1%)	651 (10.5%)
Non-Hispanic	5,193 (89.1%)	5,277 (84.8%)
Missing	338 (5.8%)	294 (4.7%)
**Insurance Status** (N(%))		
Commercial^+^	1,512 (26.4%)	1,944 (31.6%)
Commercial and Public^+^	195 (3.4%)	237 (3.9%)
Medicaid^+^	1,494 (26.1%)	1,142 (18.6%)
Medicare^+^	1,767 (30.8%)	2,165 (35.2%)
Medicare and Medicaid^+^	397 (6.9%)	329 (5.4%)
Self-Pay	356 (6.2%)	313 (5.1%)
Other	8 (0.1%)	13 (0.2%)

Missing values that exceed 5% are presented as unweighted percentages.

*American Indian or Alaskan Native (AI/AN).

**Native Hawaiian or other Pacific Islander (NHPI).

+Demographic characteristics with p-values ≤ 0.05, indicating a statistically significant difference between CHB cases and comparators.

The distribution of demographic, socioeconomic, and health characteristics of patients differed slightly across analytic stages of cohort construction. S2 Table presents the characteristics of the entire patient population prior to applications of exclusion criteria, stratified by history of CHB. S3 Table summarizes characteristics among patients who were omitted from the final study population, also stratified by history of CHB. Compared to the final analytic cohort, excluded patients were more likely to be male (66.9% vs 60.4%), White (45.0% vs. 31.6%), and insured through Medicaid (38.4% vs. 26.1%) (S3 Table).

### Geographic distribution of CHB in the Puget sound region

**Fig 2:**
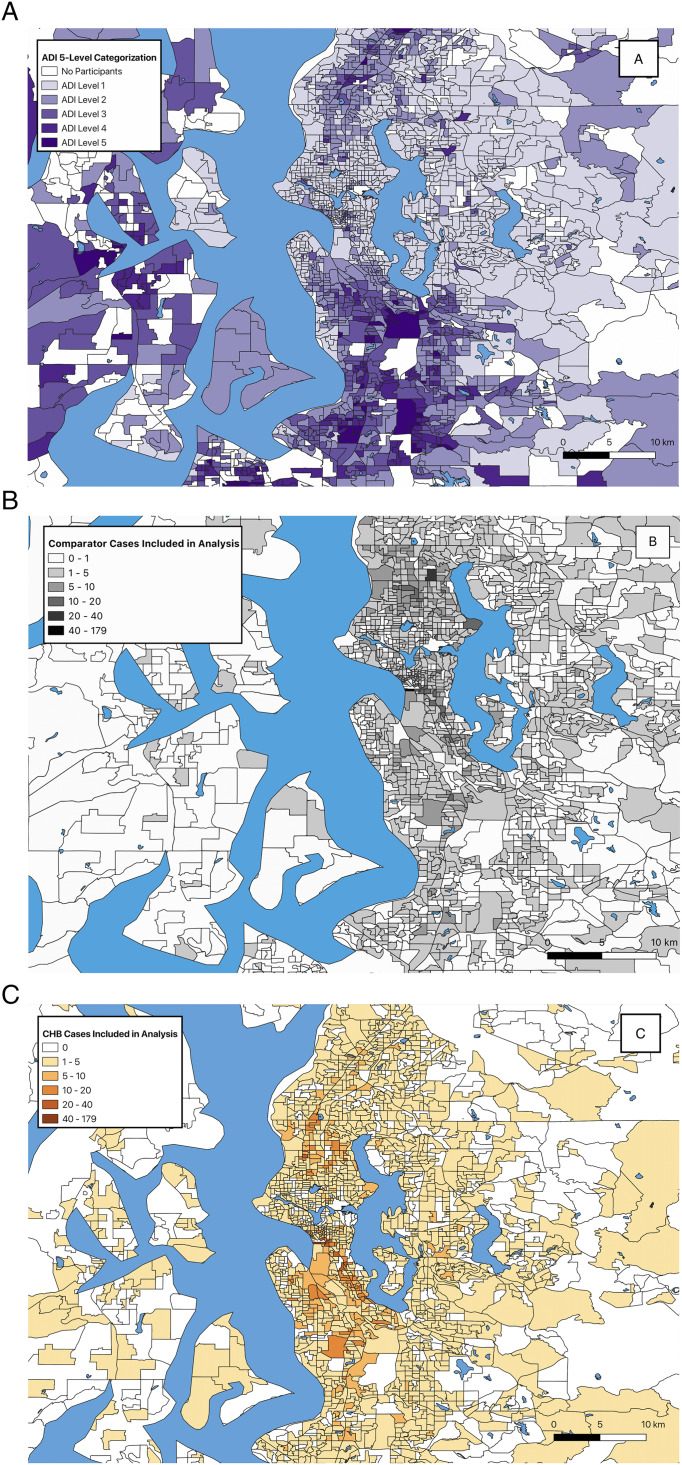
Geographic distribution of census block groups in the Puget Sound region* of Washington State. **(A)** 5-Level Categorization of the Area Deprivation Index (ADI) by census block group, with higher values indicating greater neighborhood disadvantage. **(B)** Distribution of individuals in the comparator group across census block groups. **(C)** Distribution of chronic hepatitis B (CHB) cases among census block groups. *To protect patient confidentiality, only census block groups in the Puget Sound region are shown, although individuals in the analyzed study population reside throughout Washington State.

Fig 2A, 2B and 2C present the distribution of the 5-level ADI, the number of comparator group cases by census block group included in the analysis, and the number of CHB cases by census block group in the Puget Sound region. The Seattle metropolitan area is predominantly composed of census block groups with low levels of social deprivation (ADI levels 1 and 2). Social deprivation increased in areas south of Seattle; however, census block groups with high levels of deprivation remained relatively sparse throughout the region. The highest concentration of individuals from the comparator cohort was in North Seattle and along the Interstate-5 (I-5) corridor. Individuals with CHB were primarily distributed along the I-5 corridor and in census block groups south of Seattle.

### ADI 5-level distribution and chronic HBV

Table 2 presents the association between the 5-level ADI score distribution and the odds of CHB. Overall, the distribution of ADI scores appeared similar between individuals in the comparator and CHB case groups based on the descriptive comparison of percentages. Among those with CHB, 31.5% were in the least deprived category, and 5.7% were in the most deprived category, whereas those in the comparator group had comparable proportions of 36.7% and 6.2%, respectively. In the unadjusted analysis, only individuals in ADI categories 2, (OR: 1.38, 95% CI: 1.26–1.50) 3, (OR: 1.17, 95% CI: 1.05 and 1.30) and 4 (OR: 1.17, 95% CI: 1.04–1.33) demonstrated a higher odds of CHB diagnosis compared to individuals in the least deprived ADI category (ADI 1, reference group).

**Table 2:** Association between ADI Five-Level Categorization and History of Chronic Hepatitis B, University of Washington Medicine 2018 - 2023: Unadjusted and Adjusted Odds Ratios and 95% Confidence Intervals.

ADI Category	History of Chronic Hepatitis B	No Chronic History of Hepatitis B Comparator	Unadjusted Odds Ratio (95% CI)	Adjusted* Odds Ratio (95% CI)
1	1,836 (31.5%)	2,286 (36.7%)	Reference	Reference
2	1,964 (33.7%)	1,778 (28.6%)	1.38 (1.26, 1.50)	1.29 (1.16, 1.43)
3	971 (16.7%)	1,034 (16.6%)	1.17 (1.05, 1.30)	1.13 (1.00, 1.28)
4	623 (10.7%)	661 (10.6%)	1.17 (1.04, 1.33)	1.30 (1.12, 1.50)
5	335 (5.7%)	384 (6.2%)	1.09 (0.93, 1.27)	1.69 (1.39, 2.05)

*Adjusted for age, sex, race, ethnicity, and insurance status.

After adjusting for age, sex, race, ethnicity, and insurance status, we observed a statistically significant association between ADI and CHB, specifically, for ADI categories 2, 4, and 5. The odds of CHB slightly increased with increasing ADI score: ADI category 2 vs. 1 (adjusted OR: 1.29, 95% CI: 1.16–1.43); ADI category 4 vs. 1 (adjusted OR: 1.30, 95% CI: 1.12–1.50); and ADI category 5 vs. 1 (adjusted OR: 1.69, 95% CI: 1.39–2.05). The association for ADI category 3 was attenuated and no longer statistically significant (adjusted OR: 1.13, 95% CI: 1.00–1.28), suggesting that the unadjusted association may be explained in part by differences in individual sociodemographic characteristics.

### Sensitivity analysis of ADI distribution and CHB odds ratios

In the 2-level ADI categorization (low vs. high), there was no significant association between ADI and CHB (unadjusted OR: 0.97, 95% CI: 0.89–1.05) (Table 3). After adjusting for age, sex, race, ethnicity, and insurance status, a statistically significant association was observed (adjusted OR: 1.15, 95% CI: 1.04–1.27), where the odds of CHB diagnosis were 15% higher in the population with high social deprivation compared to participants with low social deprivation.

**Table 3:** Sensitivity Analysis: Association Between ADI Categorization and History of Chronic Hepatitis B Odds Ratios, University of Washington Medicine 2018-2023.

ADI Categorization	Category	History of Chronic Hepatitis B	No History of Chronic Hepatitis B Comparator	Unadjusted Odds Ratio (95% CI)	Adjusted* Odds Ratio (95% CI)
**2 Level Categorization**	Low (Ref)	4,346 (74.5%)	4,620 (74.3%)	Reference	Reference
	High	1,383 (23.7%)	1,523 (24.6%)	0.97 (0.89, 1.05)	1.15 (1.04, 1.27)
**5 Level Categorization**	1	1,836 (31.5%)	2,286 (36.7%)	Reference	Reference
	2	1,964 (33.7%)	1,778 (28.6%)	1.38 (1.26, 1.50)	1.29 (1.16, 1.43)
	3	971 (16.7%)	1,034 (16.6%)	1.17 (1.05, 1.30)	1.13 (1.00, 1.28)
	4	623 (10.7%)	661 (10.6%)	1.17 (1.04, 1.33)	1.30 (1.12, 1.50)
	5	335 (5.7%)	384 (6.2%)	1.09 (0.93, 1.27)	1.69 (1.39, 2.05)
**10 Level Categorization**	1	1,146 (18.7%)	862 (15.0%)	Reference	Reference
	2	1,140 (18.6%)	974 (17.0%)	1.14 (1.00,1.28)	1.16 (1.00, 1.34)
	3	898 (14.6%)	956 (16.7%)	1.42 (1.25, 1.61)	1.36 (1.17, 1.57)
	4	880 (14.3%)	1008 (17.6%)	1.52 (1.34, 1.73)	1.43 (1.23, 1.65)
	5	556 (9.1%)	546 (9.5%)	1.31 (1.13, 1.51)	1.25 (1.05, 1.49)
	6	478 (7.8%)	425 (7.4%)	1.18 (1.01, 1.38)	1.18 (0.98, 1.42)
	7	386 (6.3%)	377 (6.6%)	1.30 (1.10, 1.53)	1.40 (1.15, 1.70)
	8	275 (4.5%)	246 (4.3%)	1.19 (0.98, 1.44)	1.40 (1.11, 1.76)
	9	226 (3.7%)	201 (3.5%)	1.18 (0.96, 1.46)	1.69 (1.32, 2.17)
	10	158 (2.6%)	134 (2.3%)	1.13 (0.88, 1.44)	2.07 (1.51, 2.82)

*Adjusted for age, sex, race, ethnicity, and insurance status.

We observed a variable association across the 10-level categorization of neighborhood social deprivation (Table 3). In the unadjusted models, certain mid-range categories of social deprivation were associated with higher odds of CHB diagnosis, with the highest unadjusted odds rations observed in categories 3 (OR: 1.42, 95% CI: 1.25, 1.61), 4 (OR: 1.52, 95% CI: 1.34, 1.73), and 5 (OR: 1.31, 95% CI: 1.13, 1.51). Although categories 9 and 10 represented the most deprived neighborhoods, their unadjusted odds ratios were not statistically significant (OR: 1.18, 95% CI: 0.96–1.44, and OR: 1.13, 95% CI: 0.88–1.44, respectively).

After adjusting for age, sex, race, ethnicity, and insurance status, the association between social deprivation and CHB across the 10-level categorizations followed an overall upward trend. In the most versus the least deprived category comparison (ADI category 10 vs. 1), we observed two-fold higher odds of CHB in the most socially deprived group (adjusted OR: 2.07, 95% CI: 1.51–2.82).

## Discussion

We found an association between neighborhood-level social deprivation and CHB diagnosis within a large, urban healthcare system in Washington state. Adjusted models suggested that individuals in the most deprived neighborhoods (ADI 5) could have up to double the odds of a positive CHB diagnostic test compared to individuals in the least deprived neighborhoods (ADI 1). Our sensitivity analysis, which used a 10-level ADI categorization, suggested an overall positive relationship with higher odds of a positive CHB diagnostic test with increasing neighborhood-level social deprivation.

To our knowledge, this is the first study to use the ADI to assess the relationship between social deprivation and the distribution of CHB in the United States. ADI has been used to explore the geographic burden of conditions ranging from cancer care and diabetes to COVID-19 and discoid lupus [7–10]. A study conducted in Louisiana found that higher neighborhood social deprivation, measured by ADI, was associated with increased incidence of HCC. Individuals living in the highest quartile of deprivation had an incidence rate of 9.26 cases per 100,000, compared with 5.80 among those in the lowest quartile [18]. Since CHB is a major risk factor for HCC [19,20], these findings support our observations and suggest that structural risk factors may play a role in hepatitis B-related outcomes. In contrast to our results, the survival disparity observed in the Louisiana study was attenuated after adjusting for individual clinical factors [18]. One potential reason that our study noted an increase in the association after adjusting for individual-level factors could be the larger number of individuals residing in Seattle who originate from an HBV-endemic region, compared to Louisiana. In this context, individual-level factors like sex, race, ethnicity, and insurance may confound the association by masking the role of neighborhood-level social deprivation. Once these factors are accounted for, a true contribution of neighborhood disadvantage becomes apparent and thus strengthens the observed association.

The association between higher levels of neighborhood social deprivation and increased odds of a positive CHB diagnostic test is likely influenced by a complex interplay of structural and social determinants of health. In the United States, CHB is disproportionately prevalent among immigrant and first-generation populations from hepatitis B endemic regions, with studies estimating that over 80% of individuals living with CHB acquired the infection in their country of birth [2,21,22]. Individuals from hepatitis B endemic regions often settle in communities characterized by shared cultural and linguistic backgrounds that are frequently located in socioeconomically disadvantaged neighborhoods [12–14]. These areas, reflected by higher ADI scores, are shaped by systemic factors such as limited affordable housing, income inequity, reduced access to education, and limited employment, which may further impede access to hepatitis B screening, vaccination, and linkage to care through intersecting structural and social barriers [12,23].‌‌‌‌

Additionally, neighborhoods with higher levels of social deprivation often experience greater burdens of substance use disorders, including injection drug use, which may further contribute to hepatitis B transmission; however, this pathway was not directly measured in our study and should be interpreted as a potential contributing mechanism rather than a demonstrated causal pathway [24–26].

The observed association between CHB diagnosis and neighborhood-level social deprivation may be attenuated due to characteristics of the study population. Most of the study population resided in Western Washington, a region that includes more affluent areas based on the state-level ADI decile ranking system. As shown in Fig 2, the study cohort (CHB and comparator groups) was primarily located in areas with low social deprivation in North Seattle (ADI levels 1 and 2). The limited ADI variability may weaken the strength of the observed association. As a result, this study may underrepresent individuals without healthcare access, who may be both more socioeconomically disadvantaged and more likely to have undiagnosed CHB.

Our findings should be interpreted with caution due to some limitations. First, given the observational, case-control design and reliance on EHR data, the associations observed in this study cannot be interpreted as causal relationships. Second, the study population was restricted to individuals receiving care within the UW Medicine healthcare system, which does not capture all CHB cases in Washington state and may not be representative of the broader population. However, we found that individuals diagnosed with CHB were more likely to be male, identify as Asian, Black or African American, or Native Hawaiian/Pacific Islander, and to have public insurance coverage, which aligns with previously described characteristics [1,3–6], suggesting that our population was reasonably representative. Second, the comparator group, although frequency-matched to CHB cases by clinical department and time of encounter, was drawn from a population seeking care for unrelated reasons. While this approach aimed for control of healthcare access and utilization, it may have introduced selection bias, as care-seeking individuals may differ systematically from the general population in both health status and socioeconomic characteristics. Misclassification is also possible if some individuals in the comparator group had undiagnosed CHB, given that HBV screening is not universally performed in clinical practice. However, any such misclassification would be expected to be non-differential with respect to outcomes and therefore would likely attenuate observed associations toward the null rather than introduce systemic bias. Selection bias may also have been introduced through the use of billing addresses to geocode participants. These addresses may not accurately reflect current living situations, especially among individuals with unstable housing, and those with outdated or invalid addresses were excluded from the study. Additionally, while the ADI is a validated measure of neighborhood socioeconomic status, it does not capture individual-level variation or specific social determinants of health, such as immigration status, language barriers, or cultural factors, all of which may influence CHB risk and engagement in care. Finally, hepatitis B screening was not universal during the period of study, typically performed only when risk factors or potential exposure were present, which could have contributed to underdiagnosis and additional selection bias.

Our study has strengths, including the use of a large and demographically diverse sample drawn from one of the region’s largest healthcare systems. The availability of electronic health record data enabled the identification of CHB cases using laboratory-confirmed results and facilitated the construction of a comparator group matched by healthcare utilization and time period.

In conclusion, we found an association between neighborhood-level social deprivation and an increased odds of a positive CHB diagnostic test, particularly when using more granular measures of deprivation. While individuals with CHB were more likely to be male, identify as Asian, Black or African American, or Native Hawaiian Pacific Islander, and be publicly insured, these individual-level factors only partially explained the observed disparities. Structural and contextual neighborhood factors may help identify communities at higher risk for CHB in ways that are not captured by individual-level demographic characteristics alone.

The use of geographic indicators such as the Area Deprivation Index (ADI) may assist public health practitioners in identifying underserved communities—not only to enhance case detection and linkage to care, but also to inform efforts aimed at understanding and addressing the systemic barriers that impede adequate screening and treatment for chronic hepatitis B. Effective public health interventions for outreach, education or testing should actively engage local communities, ensuring that strategies are responsive to the unique social and cultural realities of the place where people live. Further research is needed to better understand the mechanisms underlying the observed association, including how neighborhood disadvantage interacts with cultural and systemic barriers to care. Future studies should also incorporate alternative measures of social deprivation and include more socioeconomically and geographically diverse populations to validate these findings. Ultimately, integrating neighborhood context into public health planning could enhance efforts to reduce the burden of CHB and address long-standing health disparities.

## Supporting information

S1 Table Variables included in the composite Area Deprivation Index Score assigned to individual U.S. census block groups [25]. (PDF)

S2 Table Demographic, socioeconomic, and health characteristics among total University of Washington Medicine patients, 2018–2023, prior to exclusion criteria, segregated by history of chronic Hepatitis B. (PDF)

S3 Table Demographic, socioeconomic, and health characteristics among University of Washington Medicine, 2013–2023, patients omitted from the final study population segregated by history of chronic Hepatitis B. (PDF)
