# Supplementary material for: Association between neighborhood disadvantage and chronic hepatitis B in the central Puget Sound region of Washington, 2018 to 2023
Source: PLoS One. 2026 Jun 15;21(6):e0349563. doi: 10.1371/journal.pone.0349563 (PMC13268147; doi:10.1371/journal.pone.0349563)
Supplement: S1 Table — (PDF) [file pone.0349563.s001.pdf]

S1 Table. Variables included in the composite Area Deprivation Index Score assigned to individual U.S. census block groups [24].

Domain	Variable
Education	% Population aged 25 years or older with less than 9 years of education<br>% Population aged 25 years or older with at least a high school diploma<br>% Population aged 16 years or older in white-collar occupations.
Income/Employment	Median family income in U.S. dollars<br>Income disparity<br>% Families below federal poverty level<br>% Population below 150% of federal poverty level<br>% Civilian labor force population aged 16 years and older who are unemployed
Housing	Median home value in U.S. dollars<br>Median gross rent in U.S. dollars<br>Median monthly mortgage in U.S. dollars<br>% Owner-occupied housing units
Household characteristics	% Single-parent households with children younger than 18<br>% Households without a motor vehicle<br>% Households without a telephone<br>% Households with more than 1 person per room<br>% Occupied housing units without complete plumbing
