# Supplementary material for: Association between neighborhood disadvantage and chronic hepatitis B in the central Puget Sound region of Washington, 2018 to 2023
Source: PLoS One. 2026 Jun 15;21(6):e0349563. doi: 10.1371/journal.pone.0349563 (PMC13268147; doi:10.1371/journal.pone.0349563)
Supplement: S2 Table — (PDF) [file pone.0349563.s002.pdf]

S2 Table. Demographic, socioeconomic, and health characteristics among total University of Washington Medicine patients, 2018-2023, prior to exclusion criteria, segregated by history of chronic Hepatitis B.

**Characteristic**	**History of Chronic Hepatitis B N = 6,500**	**No History of Chronic Hepatitis B Comparator N = 6,955**
**Age** (Mean (SD))	56 (14.4)	55 (17.6)
**Sex Assigned at Birth (N(%))**		
Female	2,528 (38.9%)	3,262 (46.9%)
Male	3,970 (61.1%)	3,693 (53.0%)
**Race** (N(%))		
AIAN	79 (1.2%)	132 (1.9%)
Asian	2,127 (32.7%)	773 (11.1%)
Black or African American	1,467 (22.6%)	853 (12.3%)
Multi-Racial	134 (1.6%)	134 (1.9%)
NHPI	161 (2.5%)	63 (0.9%)
White	2,157 (33.1%)	4,597 (66.1%)
Other	28 (0.4%)	46 (0.7%)
Missing	357 (5.8%)	357 (5.1%)
**Ethnicity (Hispanic) (N(%))**		
Hispanic	333 (5.1%)	729 (10.5%)
Non-Hispanic	5,781 (88.9%)	5,882 (84.6%)
Missing	386 (5.9%)	344 (4.9%)
**Insurance Status** (N(%))		
Commercial	1,628 (25.0%)	2,124 (30.5%)
Commercial and Public	216 (3.3%)	269 (3.9%)
Medicaid	1,790 (27.5%)	1,370 (19.7%)
Medicare	1,986 (30.6%)	2,425 (34.9%)
Medicare and Medicaid	461(7.1%)	375 (5.4%)
Self-Pay	409 (6.3%)	379 (5.4%)
Other	10 (0.2%)	13 (0.2%)

Missing values that exceed 5% are presented as unweighted percentages
*American Indian or Alaskan Native (AI/AN)
**Native Hawaiian or other Pacific Islander (NHPI)
