# Supplementary material for: Association between neighborhood disadvantage and chronic hepatitis B in the central Puget Sound region of Washington, 2018 to 2023
Source: PLoS One. 2026 Jun 15;21(6):e0349563. doi: 10.1371/journal.pone.0349563 (PMC13268147; doi:10.1371/journal.pone.0349563)
Supplement: S3 Table — (PDF) [file pone.0349563.s003.pdf]

S3 Table: Demographic, socioeconomic, and health characteristics among University of Washington Medicine, 2013-2023, patients omitted from the final study population segregated by history of chronic Hepatitis B.

**Characteristic**	**History of Chronic Hepatitis B N = 771**	**No History of Chronic Hepatitis B Comparator N = 812**
**Age** (Mean (SD))	55 (13.9)	54 (17.27)
**Sex Assigned at Birth** (N(%))		
Female	254 (32.9%)	353 (43.4%)
Male	516 (66.9%)	456 (56.5%)
**Race (**N(%))		
AIAN	20 (2.6%)	25 (3.1%)
Asian	154 (20.0%)	52 (6.4%)
Black or African American	110 (20.0%)	110 (13.5%)
Multi-Racial	14 (1.8%)	17 (2.1%)
NHPI	25 (3.2%)	8 (1.0%)
White	347 (45.0%)	555 (68.3%)
Other	3 (0.4%)	4 (0.5%)
Missing	54 (7.0%)	41 (5.0%)
**Ethnicity (Hispanic)** (N(%))		
Hispanic	38 (4.9%)	88 (10.8%)
Non-Hispanic	680 (88.1%)	672 (82.8%)
Missing	53 (6.9%)	52 (6.4%)
**Insurance Status** (N(%))		
Commercial	116 (15.0%)	180 (22.2%)
Commercial and Public	21 (2.7%)	32 (4.0%)
Medicaid	296 (38.4%)	228 (28.1%)
Medicare	219 (28.4%)	260 (32.0%)
Medicare and Medicaid	64 (8.3%)	46 (5.7%)
Self-Pay	53 (6.9%)	66 (8.1%)
Other	0 (0.0%)	0 (0.0%)

Missing values that exceed 5% are presented as unweighted percentages

*American Indian or Alaskan Native (AI/AN)

**Native Hawaiian or other Pacific Islander (NHPI)
